# Valorization of *Manihot esculenta* peel from environmental pollutant to sustainable engineering solutions for a cleaner future

**DOI:** 10.1007/s11356-024-35621-8

**Published:** 2024-11-29

**Authors:** Festus Ben

**Affiliations:** 1 Centre for Nanoengineering and Advanced Materials, Department of Metallurgy, University of Johannesburg, Johannesburg, South Africa; 2 Centre for Materials Research and Development, Department of Physics, Federal Polytechnic Ede, Ede, Nigeria

**Keywords:** *Manihot esculenta*, Cleaner production, Environmental pollution, Sustainable engineering material, Environmental remediation, Valorization, Agro-waste management

## Abstract

As efforts intensify to address the environmental impact of agricultural waste, the valorization of *Manihot esculenta* peel (MEP) for sustainable engineering applications presents a unique opportunity to repurpose this class of agricultural waste to achieve environmental sustainability development goals while promoting socio-economic development of this pollutant. The inherent properties of MEP, such as its richness in carbohydrates and cellulose, make it a useful raw material for producing biofuels, bioethanols, biocomposites, and other sustainable engineering materials. Its resilience to adverse environmental conditions also makes MEP well-suited for cultivation in diverse agroecological settings, further enhancing its appeal as a sustainable resource. While existing review articles provide valuable insights into *Manihot esculenta* peel utilization across various industries, they often overlook the comprehensive valorization of *Manihot esculenta* for sustainable engineering applications, creating a notable knowledge gap. Through a systematic examination of innovative approaches documented in the literature, this research seeks to bridge this gap by elucidating strategies for repurposing cassava waste into valuable engineering materials to mitigate environmental pollution and promote sustainable resource utilization. By synthesizing existing research and identifying key research gaps, this study advances the understanding of *Manihot esculenta* peel’s potential as a sustainable material and facilitates the transition toward greener engineering practices.

## Introduction

In the swiftly evolving global landscape, characterized by diverse environmental challenges, sustainability has evolved as a paramount consideration in addressing issues stemming from environmental degradation and resource depletion. Sustainable engineering has been underscored as pivotal to fostering socio-economic growth while reiterating the need for environmental protection (Thorpe 2018). As such, sustainable engineering stands as a key strategy for optimizing environmental impact by promoting efficient resource use and minimizing depletion, focusing on developing technologies, processes, and materials that reduce environmental harm and enhance resilience against climate change. Amidst these imperatives, the issue of environmental pollution from agricultural (agro) waste has become increasingly urgent.

The FAO (Food and Agriculture Organization) reported a 14% increase in world post-harvest waste in 2019 (FAO 2022), escalating to a 17% increase by 2021 (Marchant 2021), highlighting the severity of the issue. The United Nations Environmental Programme (UNEP) estimated the yearly agro-waste production to be around 931 million tonnes, making it the third-largest origin of global greenhouse gas emissions (UNEP 2021). Agro-waste is, therefore, one of the leading causes of environmental pollution, resulting in ecosystem degradation. Recognizing the urgency, the United Nations (UN) has targeted the reduction of agro-waste through Sustainable Development Goal (SDG) 12.3, aiming to cut down agro-waste loss by 2030 (UN 2022). Repurposing agricultural waste for sustainable engineering applications emerges as a promising solution for a cleaner environmental future and for mitigating environmental pollution. This urgency has given rise to innovative approaches such as valorization, which holds significant importance in effective sustainable engineering and environmental remediation practices.

Valorization involves transforming waste materials into useful and valuable products, thereby conserving natural resources and reducing environmental pollution. Agro-waste, encompassing both plant and animal residues, can undergo valorization through various engineering techniques to produce materials tailored for specific applications. Numerous studies have documented the valorization of agro-waste products, including the fabrication of composites with enhanced mechanical, tribological, and structural properties (Edhirej et al. 2021; Joseph and Babaremu 2019; Jullanun and Yoksan 2020; Vinod et al. 2020), utilization as raw materials in building and construction projects (Jayanthi et al. 2023; Mendívil et al. 2017; Rao et al. 2021; Ricciardi et al. 2020), and integration into remediation processes (Elemike et al. 2022; Kapahi and Sachdeva 2019). Through valorization, agro-waste materials undergo processes such as extraction, purification, modification, and formulation to enhance their properties for specific applications. The valorization of agro-waste has contributed to environmental protection when used as biosorbents for remediating heavy metals and minimizing landfill disposals (Simona et al. 2018). It has also led to the development of innovative engineering materials with applications spanning industries such as automotive, packaging, electronics, aerospace, and water treatment (Aigbodion et al. 2018; Anawar et al. 2015; Elemike et al. 2022; Martinaud et al. 2024; Valencia et al. 2021). This transformative process not only reduces environmental pollution but also creates opportunities for the development of sustainable solutions across various sectors.

Agricultural wastes encompass both animal byproducts, including fats, edible offal, meat, skins, hides, shells, bones, and feathers, as well as plant residues derived from food crops like dates, bananas, pineapples, vegetables, oil crops, fiber crops, sugar crops, tree nuts, pulses, cereals, and tubers and roots such as yams, potatoes, cassava, and taro (Ben and Olubambi 2024a; FAO 2020; Singh et al. 2022). This research focuses primarily on *Manihot esculenta* (ME), commonly known as cassava, recognized for its starchy tuberous roots serving as a staple food source worldwide and its richness in carbohydrates and cellulose (Adebayo 2023; Okoli 2020). Of particular interest are the peels of ME, considered a significant environmental pollutant (Ben and Olubambi 2024b). Various studies have explored strategies to repurpose the *Manihot esculenta* peels (MEP) for sustainable engineering applications (Awokoya et al. 2016; Hartini et al. 2015; Mohd-asharuddin et al. 2017; Nida et al. 2022; Odeyemi et al. 2023; Ogunbode et al. 2023a; Olatokunbo et al. 2018; Olumide et al. 2022; Sudaryanto et al. 2006; Versino et al. 2015). For instance, Versino et al. (2015) demonstrated an enhancement in the mechanical constraint properties of thermoplastic starch (TPS) biocomposites by incorporating MEP alongside bagasse as a filler material. Awokoya et al. (2016) investigated the efficacy of MEP as a biosorbent for removing copper and nickel ions from aqueous solutions, achieving efficient performance. Additionally, enhancement in the physical properties of MMCs (metal matrix composites) reinforced MEP hybrid variants in ash form has also been reported by (Ogunbode et al. 2023a; Oladele et al. 2020; Olaniran et al. 2021). Olatokunbo et al. (2018) observed that *Manihot esculenta* peel ash (MEPA) shares similar chemical constituents with cement, proposing its use as a suitable substitute for cement in light construction activities.

The substantial progress documented in the experimental utilization of MEP particulates presents a unique opportunity to repurpose them for sustainable engineering applications, contributing to a cleaner environment. This progress has spurred the emergence of several review articles examining *Manihot esculenta’s* potential as a sustainable raw material for addressing crucial ecological concerns (Adekunle et al. 2016; Andrade et al. 2021; Morgan and Choct 2016; Okudoh et al. 2014; Präger et al. 2019; Santos et al. 2015; Senanu et al. 2023; Tumwesigye et al. 2016; Wang et al. 2022; Zhang et al. 2016). These review articles explored topics ranging from the use of MEP to produce activated carbon (Edhirej et al. 2021; Kayiwa et al. 2021a), to utilizing MEP wastes for biogas production (Andrade et al. 2021; Okudoh et al. 2014; Zhang et al. 2016), and the chemical modification of MEP starch to improve its functional properties (Ifeanyi Charles 2020; Raudhah et al. 2023; Senanu et al. 2023; Wang et al. 2022). Additionally, the reviews explored the potential of MEP biomass for sustainable energy production (Andrade et al. 2021; Otun et al. 2023; Präger et al. 2019), its role in biobased packing materials (Tumwesigye et al. 2016), and its nutritive value in poultry and animal diets (Devi and Diarra 2021). Despite the breadth of topics covered, a significant knowledge gap exists in the literature regarding the holistic valorization of ME residues for sustainable engineering applications, particularly in environmental remediation and circular economy principles. While some reviews touched upon aspects of ME waste utilization, such as biogas production and biomass sources for energy supply, there is limited focus on the comprehensive valorization of ME residues across multiple sectors.

In addressing this gap, this study investigates *Manihot esculenta* residues as a potential sustainable engineering material, demonstrating the environmental and socio-economic utility of transforming *Manihot esculenta* peels (MEP) from agricultural waste into valuable engineering materials, presenting a viable pathway for sustainable resource use. By promoting waste valorization, this research supports circular economy principles and contributes to cleaner production practices, reducing dependency on non-renewable resources. Specifically, it explores ME peel ash (MEPA) for applications in reinforcement materials in biocomposites, concrete binders, pozzolan for soil stabilization, and pollutant remediation. Integrating insights from biotechnology, materials science, and environmental engineering, this research advances sustainable engineering for a cleaner future.

### *Manihot* esculenta

**Fig. 1:**
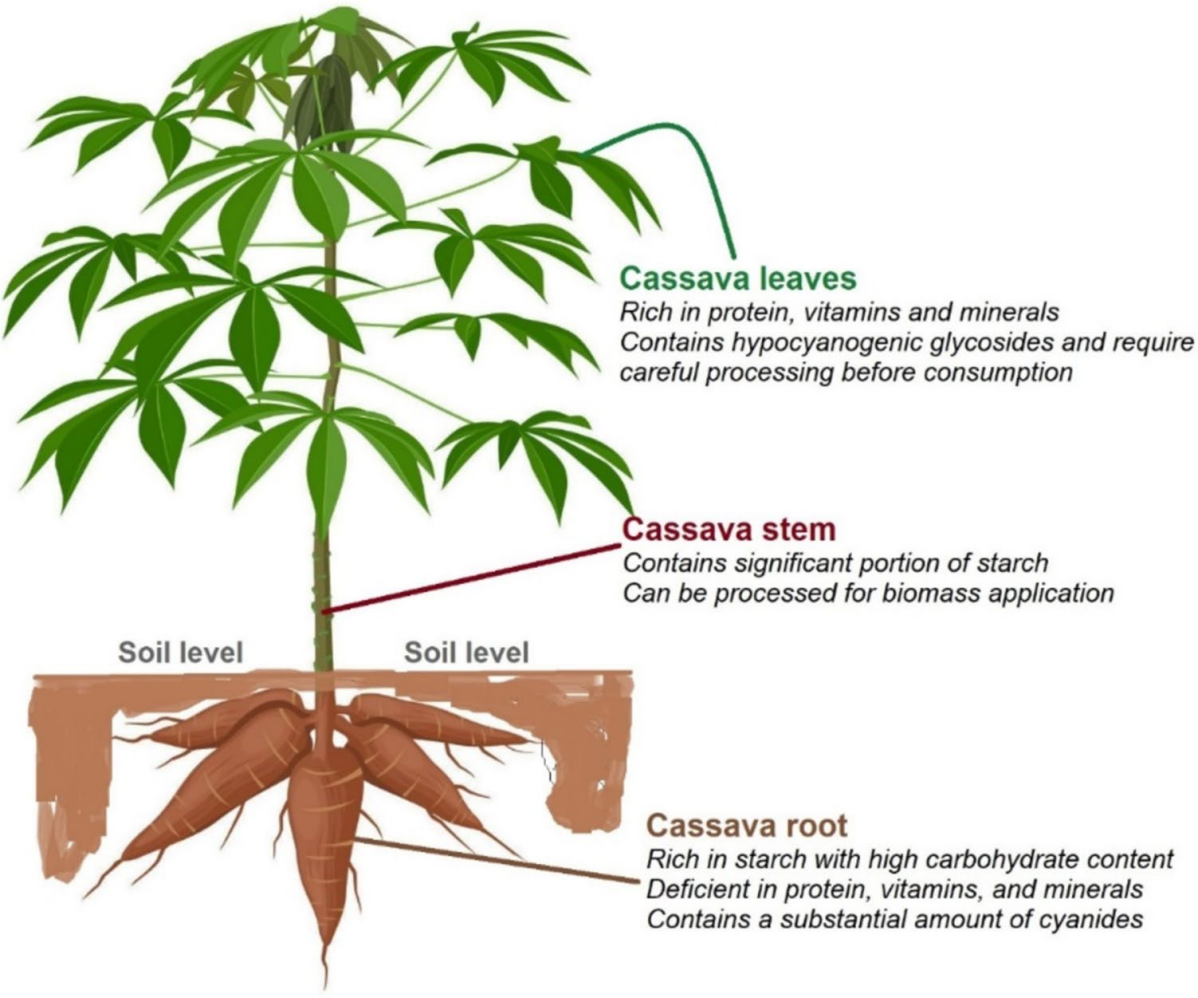
Illustration of *Manihot esculenta* depicting its various components and nutritional composition

*Manihot esculenta*, a dicotyledonous plant of the Euphorbiaceae family comprising the leaves, stems, and roots (Fig. 1), is a vital crop in tropical regions. While the leaves are rich in nutrients like vitamins, proteins, and minerals (Otun et al. 2023), the stems contain approximately 30% starch by dry mass, making them suitable for biofuel production (Zhu et al. 2015). The tuberous roots contain cyanogen glycoside, posing toxicity risks to animals, humans, and pathogens (Panghal et al. 2021; Shigaki 2016). Nonetheless, these roots are a staple food for over 900 million people globally, with water-leaching techniques employed to mitigate cyanide toxicity. The perennial crop is drought-tolerant and thrives in tropical regions with low soil nutrients and rainfall (Asogwa et al. 2017).

**Fig. 2:**
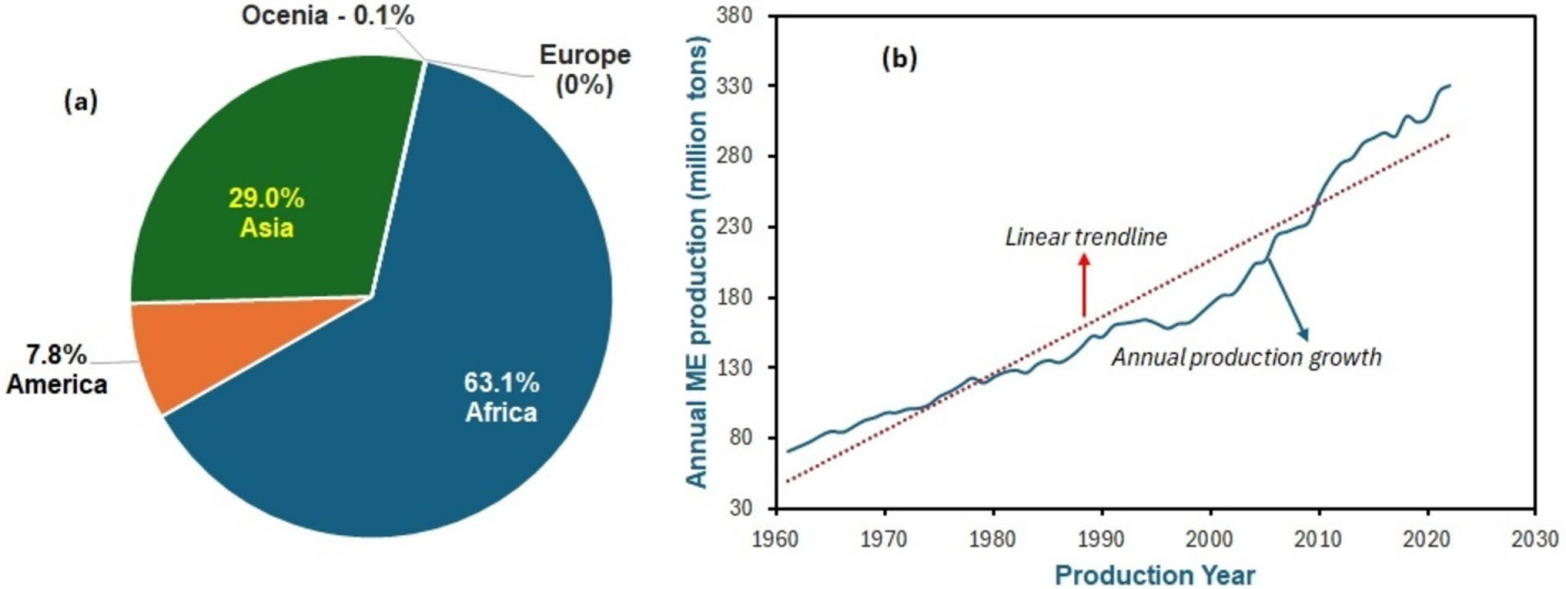
Production trends of *Manihot esculenta* depicted regionally (**a**) and annually (**b**)

Despite originating in Latin America, Africa leads in *Manihot esculenta* production at ~ 63%, Asia at 29%, and South America at ~ 8% (Fig. 2a). Global production also increased over time (Fig. 2b), reaching approximately 330 million tonnes annually (Knoema 2022), making it the third most produced crop after wheat, rice, and maize (Adebayo 2023). *Manihot esculenta* is largely produced in Nigeria, with about 18.41% contribution to worldwide production (FAO 2023). About 30% of *Manihot esculenta* is used for industrial purposes and animal feed (Caccamisi 2010). Its rich carbohydrate and mineral content make it a valuable crop for both food and biofuel industries (Ifeanyi Charles 2020; Morgan and Choct 2016).

### *Manihot esculenta* peels

The root of *Manihot esculenta* is a key global food source, but its peel, constituting about 15% of the root mass, is often discarded as agricultural waste. These peels contain cyanogenic glycosides, which release toxic hydrogen cyanide upon hydrolysis, posing significant environmental risks. Improper disposal, including burning, releases harmful gases persisting in the atmosphere for approximately 150 days (Karlsson 2004), further contributing to pollution and climate change, particularly in regions with limited waste management. Cyanide exposure from these wastes can severely affect human health, causing symptoms from dizziness to death, and also contaminates soil and water, impacting local ecosystems (Manila and Devi 2021; Jaszczak et al. 2017; Meeussen et al. 1995).

Considering that approximately 0.25 tonnes of *Manihot esculenta* peel waste is generated for every 1 ton of fresh ME root, Table 1 presents the estimated percentage of MEP waste production based on this estimate. Global *Manihot esculenta* peel waste production stands at ~ 82 million tons, with Nigeria contributing approximately 18%, or ~ 15 million tons (Table 1). This marks a substantial increase from 2014 (Ben and Olubambi 2024a) and reflects the escalating environmental threat of ME peel waste, underscoring the urgency of mitigating its environmental impact by transforming *Manihot esculenta* peels from an environmental pollutant into valuable engineering materials, promoting waste valorization as a means to support cleaner production and sustainable resource use.

**Table 1:** Global, regional, and country-specific production of *Manihot esculenta* peel wastes

Global/regional/country	Production of ME (tonnes)	Production of ME peels (tonnes)	% global production of ME peels
Global production	330,408,753.77	82,602,188.44	100.00
Production by region			
Africa	208,627,012.53	52,156,753.13	63.14
Asia	95,719,378.86	23,929,844.72	28.97
Americas	25,785,439.73	6,446,359.93	7.80
Oceania	276,922.65	69,230.66	0.08
Production by the top 20 producing countries			
Nigeria	60,835,539.96	15,208,884.99	18.41
Democratic Republic of the Congo	48,774,623.00	12,193,655.75	14.76
Thailand	34,068,005.00	8,517,001.25	10.31
Ghana	25,592,014.08	6,398,003.52	7.75
Cambodia	17,698,783.81	4,424,695.95	5.36
Brazil	17,648,564.00	4,412,141.00	5.34
Indonesia	13,574,000.00	3,393,500.00	4.11
Viet Nam	10,626,861.72	2,656,715.43	3.22
Angola	10,547,506.00	2,636,876.50	3.19
Mozambique	6,466,857.00	1,616,714.25	1.96
United Republic of Tanzania	6,354,438.68	1,588,609.67	1.92
Côte d’Ivoire	6,300,000.00	1,575,000.00	1.91
Cameroon	6,267,574.33	1,566,893.58	1.90
Malawi	6,239,912.01	1,559,978.00	1.89
India	6,213,000.00	1,553,250.00	1.88
Lao People’s Democratic Republic	5,286,000.00	1,321,500.00	1.60
China	5,049,032.62	1,262,258.16	1.53
China, mainland	5,040,474.48	1,260,118.62	1.53
Benin	4,350,053.57	1,087,513.39	1.32
Zambia	3,497,600.74	874,400.19	1.06

Source: Author’s extrapolated data from FAO (2023)

### Heat treatment strategies for *Manihot esculenta* peels

To mitigate the environmental consequences resulting from the indiscriminate disposal of *Manihot esculenta* peels, the heat treatment technique offers a promising solution for repurposing MEP wastes into sustainable raw materials for diverse applications. The heat treatment process precludes carbonization, calcination, or ashing of MEP wastes. This valorization approach, inspired by recycling principles as described by Festus et al. (2019), typically begins with the collection of *Manihot esculenta* peel wastes, which are then subjected to drying to remove moisture content, enhancing the stability and storability of MEPs while retaining nutritional and functional properties (Ben et al. 2023).

Sun-drying removes moisture from MEPs and reduces cyanide levels by approximately 90% without affecting the nutritional content (Devi and Diarra 2021). Oven drying at controlled temperatures (46–100 °C) further minimizes cyanide levels and moisture content (Nambisan 1994; Leeson and Summers 2009). Advanced methods, including vacuum, infrared, and hybrid drying, also offer efficient dehydration of MEPs, suitable for varied engineering applications (Carvalho et al. 2023).

**Fig. 3:**
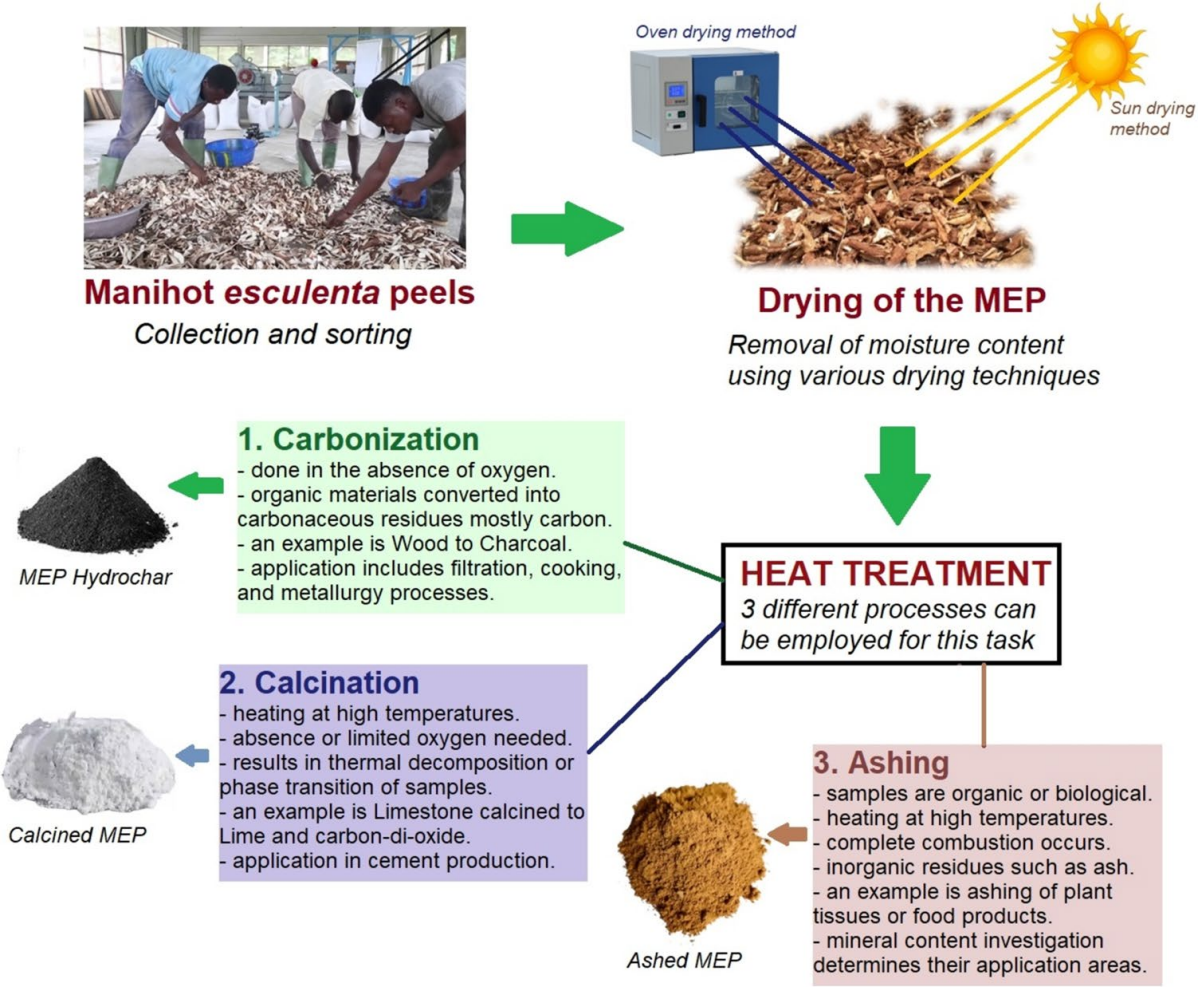
Heat treatment process for repurposing *Manihot esculenta* peels

The heat treatment strategy presented in Fig. 3 outlines a process flow, starting from MEP collection to drying, illustrating each step’s role in transforming agricultural waste into a resource-efficient material. This valorization approach aligns with sustainable production practices, presenting a viable pathway for reducing the environmental impacts of MEP disposal while producing eco-friendly, renewable materials for engineering applications.

*Carbonization* involves subjecting organic materials to heat treatment to remove moisture content and other volatile organic compounds, resulting in a carbonaceous residue with a high carbon content. This process can convert *Manihot esculenta* peels into valuable activated carbon, offering an efficient route for waste valorization. This is achieved by heating the peels to eliminate moisture and volatile compounds, yielding a carbon-rich residue with a high surface area. The activation, either chemical (e.g., using KOH, Na₂S₂O₃) or physical (CO₂ or steam), optimizes the carbon’s porosity, enhancing properties like thermal stability and electrical conductivity, ideal for supercapacitors and electric double-layer capacitors (EDLCs) used in energy storage (Ismanto et al. 2010). Research indicates that the pore nature of activated carbon is altered by factors such as pore volume, impregnation ratio, surface area, and temperature of carbonization, while activation time has no significant impact (Chmiola et al. 2006; Ismanto et al. 2010). Activated carbon materials offer advantages such as excellent thermal properties, high surface area, stability, good electrical conductivity, and resistance to causticity. Table 2 summarizes studies illustrating effective activation techniques, carbon yields, and properties of activated carbon from MEP.

**Table 2:** Carbonization techniques and properties of activated carbon from *Manihot esculenta* peels for valorization applications

Study	Activation method	Temperature (°C)	Carbon yield (%)	Key properties	Applications
Ahmadpour and Do (1997)	Chemical (KOH)	~ 500	High	Enhanced pore structure and surface area	Adsorbents, supercapacitors
Sudaryanto et al. (2006)	Physical (CO₂)	800	29	Good electrical conductivity and stability	Energy storage
Ismanto et al. (2010)	Physical and chemical activation	400–900	Varies	High thermal stability and electrical conductivity	EDLCs, carbon-based electrode materials
Zhang et al., (2021)	Chemical (H₂SO₄, HNO₃, H₂O₂)	600	Moderate	High porosity, causticity resistance	Supercapacitors, adsorbents
Amakoromo et al. (2021)	Chemical (Na₂S₂O₃, KCl)	~ 600	Moderate	Increased surface area, reduced cyanide content	Soil stabilization, pollutant remediation

*Calcination* involves heating organic residues at elevated temperatures with limited oxygen, leading to thermal decomposition or phase transition and chemical or physical changes in the material’s structure. The process typically occurs in a muffle furnace at temperatures ranging from 500 to 1000 °C for about 4 h, with optimal conditions depending on the material’s composition (Abiodun et al. 2022; Betiku and Ajala 2014; Zemnukhova et al. 2012). For *Manihot esculenta* peel (MEP), calcination has been explored for silica extraction, with studies indicating that heating MEP at 700 °C for 270 min leads to an 82% weight loss and enhanced ash content, making it suitable for use in industries such as plastics, metals, and glass (Adebisi et al. 2019; Deng et al. 2016; Vaibhav et al. 2015). Despite these promising findings, research on MEP calcination is limited compared to other agro-wastes, suggesting a need for further exploration.

*Ashing* involves heating organic materials with oxygen at high temperatures to produce inorganic residues called ash, which is essential for elemental analysis and mineral composition determination. The process typically occurs in an electric muffle furnace at temperatures between 500 and 900 °C for about 4 h, depending on specific requirements (Ben et al. 2023; Ogunbode et al. 2023a; Olaniran et al. 2021). The utilization of ash particulates derived from *Manihot esculenta* peel has been explored across various fields, including construction, biocompoites, and soil stabilization. For example, MEPA particulates have been used as reinforcement in hybrid composites (Olaniran et al. 2021), biocomposite concrete (Ogunbode et al. 2023a), and aluminum matrix composites (Ben and Olubambi 2024c). Additionally, MEPA has been explored as a partial substitute for Portland cement (Olatokunbo et al. 2018) and in soil stabilization (Edeh et al. 2014). Beyond these industrial uses, MEPA has even been applied in soap production as a replacement for traditional lye (Adaku and Melody 2013).

### Chemical and elemental analysis of heat-treated *Manihot esculenta* peels

The main objective of heat-treating agro-waste residues through carbonization, calcination, and ashing, as depicted in Fig. 3, is to improve their purity, stability, and suitability for specific applications. Studies have examined the composition and microstructural analysis of *Manihot esculenta* peels produced using these heat treatment methods. Table 3 presents the results of chemical investigations conducted on ME peels using sun/oven drying methods. The table reveals variations in ash content, ranging from 5.16 to 10.70, and dry matter, ranging from 17.90 to 92.03. Additionally, nutritive contents such as metabolizable energy, ether extract, crude protein, and fiber exhibit varying ranges, indicating the diversity in nutritional profiles. These findings indicate the potential dietary value of *Manihot esculenta* peels for livestock feeds. Moreover, standard organizations can use this information to establish quality control measures for *Manihot esculenta* peel-based feedstock products, particularly when utilized as alternatives to maize-based feedstocks.

**Table 3:** Proximate compositions of *Manihot esculenta* peels

Metabolizable energy (MJ/kg)	Dry matter	Crude protein	Crude fibre	Ether extract	Ash	Reference
11.10	-	4.20	12.70	1.40	8.70	(Dayal et al. 2018)
-	88.80	5.24	12.38	3.97	5.16	(Oladunjoye et al. 2010)
-	-	3.56	22.65	1.21	6.02	(Ndelekwute et al. 2021)
-	90.60	5.30	11.50	0.70	9.30	(Osei et al. 1990)
11.23	-	6.19	18.9	1.18	6.01	(Dairo 2011)
-	88.96	17.33	9.89	3.61	7.13	(Adesehinwa et al. 2011)
-	17.90	4.20	29.60	3.26	7.47	(Aro et al. 2010)
-	87.90	5.12	12.09	1.19	6.01	(Okechukwu et al. 2019)
-	-	7.05	55.14	0.90	10.70	(Versino et al. 2015)
11.85	88.8	2.50	5.20	0.40	5.90	(Garcia 1995)

*Oxide compositions* of heat-treated *Manihot esculenta* peel particulates analyzed using X-ray fluorescence (XRF) revealed a predominance of silica (SiO_2_) and alumina (Al_2_O_3_), with variations observed in other oxide compositions, such as hematite (Fe_2_O_3_), quicklime (CaO), magnesia (MgO), potassium feldspar (K_2_O), soda (Na_2_O), and sulfuric oxide (SO_3_) (Awolusi et al. 2023; Nwa-David et al. 2023; Ogunbode et al. 2023a; Olatokunbo et al. 2018). Table 4 summarizes the compositions from various studies, highlighting the diverse chemical profiles of MEP particulates. The variations in oxide compositions across studies are likely attributed to differences in heat treatment conditions. These variations demonstrate the material’s potential for use in composite materials, construction, and as a partial replacement for Portland cement. Further discussion of these applications is provided in the “Building and construction materials” section.

**Table 4:** Oxide composition of heat-treated *Manihot esculenta* peel particulates

SiO_2_	Al_2_O_3_	Fe_2_O_3_	CaO	MgO	Na_2_O	K_2_O	SO_3_	Reference
36.79	7.57	2.23	8.20	2.90	1.37	18.74	1.52	(Raheem et al. 2020)
59.72	11.1	1.52	8.42	5.22	2.08	6.82	0.05	(Olatokunbo et al. 2018)
58.02	12.80	1.41	8.53	5.02	0.03	7.67	2.18	(Salau et al. 2012)
39.26	16.98	14.76	5.03	2.42	0.30	4.36	4.54	(Awolusi et al. 2023)
54.86	11.42	11.14	9.30	4.74	2.18	3.56	1.00	(Ajayi et al. 2023)
61.70	12.50	2.52	9.42	6.32	0.05	6.82	2.10	(Nwa-David et al. 2023)
55.93	19.88	6.02	9.85	3.20	0.98	-	-	(Chimmaobi et al. 2020)
83.00	2.90	2.70	1.30	0.80	-	2.80	0.00	(Ogunbode et al. 2023b)
43.16	3.95	3.32	21.09	3.40	-	-	-	(Abdulwahab and Uche 2021)

**Fig. 4:**
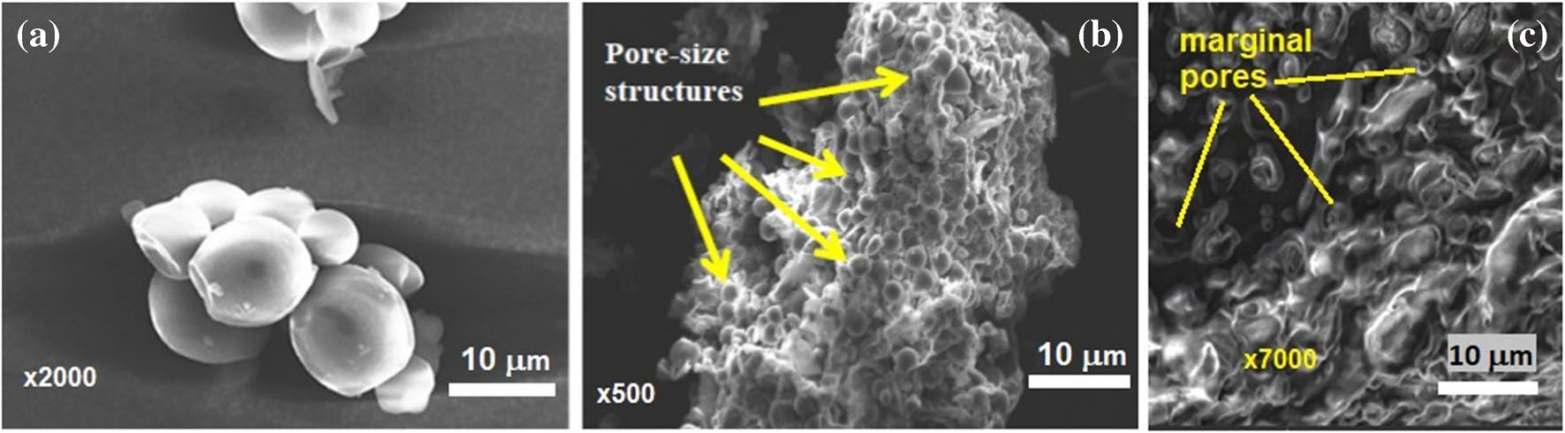
Microstrutures of *Manihot esculenta* peels surface morphology, modified from (Barati et al. 2019; Mohd-asharuddin et al. 2017; Ogunbode et al. 2023a) under CC-BY

*Surface morphology* studies of heat-treated MEPs using Scanning Electron Microscopy (SEM) provide essential insights into their structural properties, which influence their potential applications in various fields. SEM images show that MEP particulates exhibit a smooth, nonporous, round-oval morphology (Fig. 4a), making them ideal as fillers in composite materials or adsorbents for pollutants sequestration from the environment or soil conditioning (Barati et al. 2019; Ben and Olubambi 2024b; Kumar et al. 2021; Mohd-asharuddin et al. 2017; Samomssa et al. 2021). These properties also suggest MEP’s suitability for wastewater treatment, soil conditioning, and lightweight concrete applications due to its porous structure (Fig. 4b) (Awokoya et al. 2016; Mohd-asharuddin et al. 2017). At higher magnification, marginal pores were observed (Fig. 4c), which could influence concrete workability by increasing water absorption tendencies (Ogunbode et al. 2023a). Furthermore, the heterogeneous pore size distribution observed in MEP surfaces (Daud et al. 2013; Obonukut et al. 2022) affects the material’s performance in various industrial applications. Additionally, the dominance of amorphous compounds with crystalline components on the MEP surface (Belcaid et al. 2021) emphasizes the importance of processing conditions, such as activating agents, in optimizing MEP’s performance across diverse applications.

*Elemental composition* analysis of heat-treated *Manihot esculenta* peels, conducted using energy dispersive X-ray (EDX) spectroscopy, showed substantial carbon (C) content in MEP particles originating from the lignin, cellulose, and hemicellulose in MEPs (Kumar et al. 2021). Studies consistently report carbon as the dominant element, with oxygen (O), nitrogen (N), sulfur (S), calcium (Ca), phosphorus (P), and sodium (Na) appearing in varying proportions, indicating transformations during thermal processing that may impact MEP’s applicability (Ogunbode et al. 2023a; Odeyemi et al. 2023), especially in industrial contexts such as bottom-hole fluids (Idress et al. 2019). Trace elements such as aluminum (Al), silicon (Si), and potassium (K), associated with starch residues, appear less prominently but add to the compositional complexity of MEP (Idress et al. 2019; Kumar et al. 2021; Samomssa et al. 2021). This composition has significant implications for applications in supercapacitors, where carbon’s high concentration provides a cost-effective electrode material (Amakoromo et al. 2021; Ismanto et al. 2010). Moreover, understanding the emissions of combustion-related gases, such as carbon dioxide and nitrogen oxides from MEP’s carbon and nitrogen content, is crucial for assessing MEP’s environmental footprint in energy applications.

## Sustainable engineering potential of *Manihot esculenta* peels

**Fig. 5:**
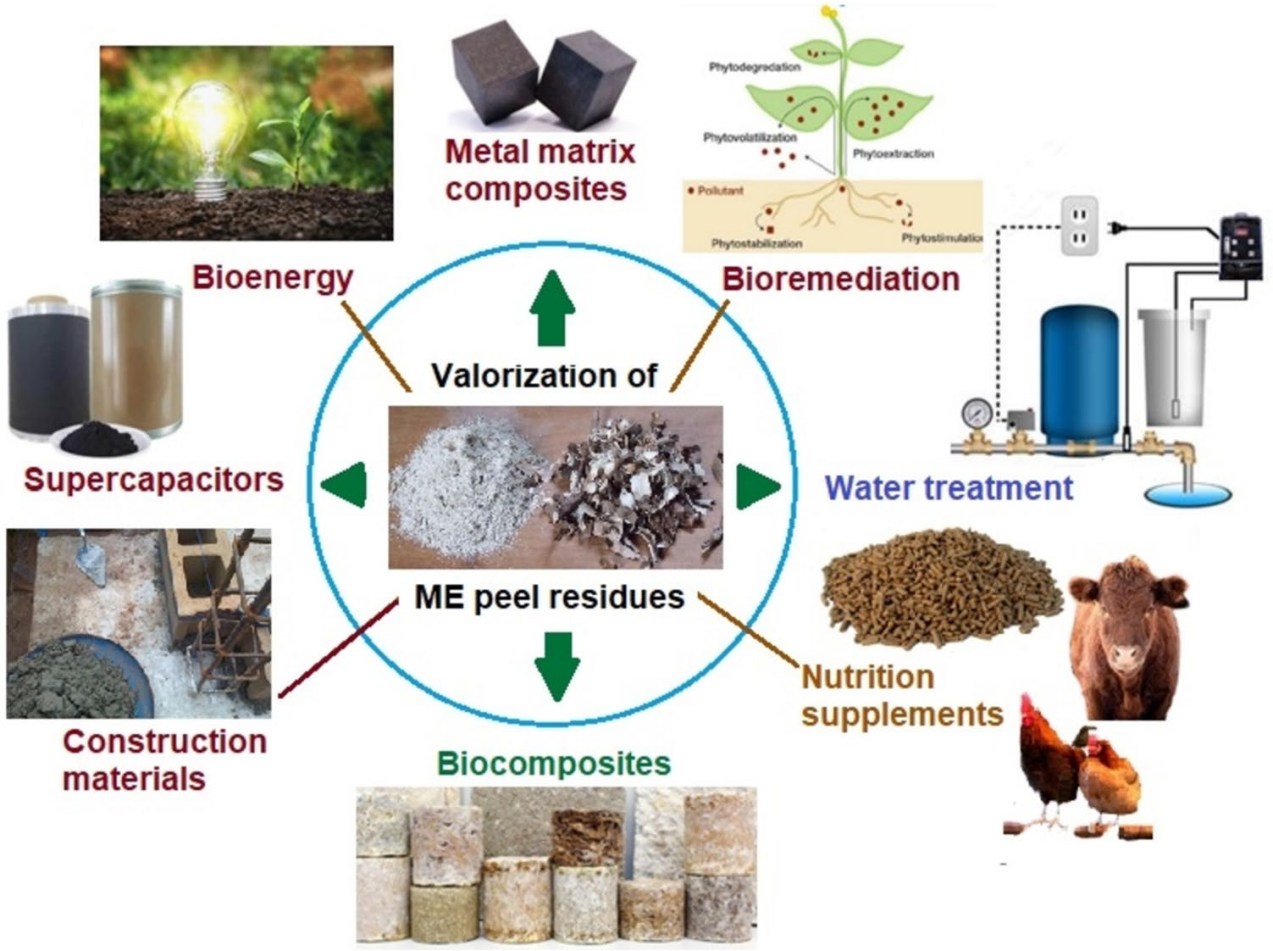
Valorization potential of ME peels for sustainability

*Manihot esculenta* peels have been repurposed by researchers for different sustainability solutions as part of attempts to address the environmental menace of this category of agro waste. Figure 5 depicts the different sustainability solutions that have been developed from MEPs, including biofuel production (Zhu et al. 2015), biogas generation (Andrade et al. 2021; Okudoh et al. 2014), livestock feed and nutrient supplements (Ifeanyi Charles 2020; Khempaka et al. 2014; Morgan and Choct 2016; Ndelekwute et al. 2021; Okechukwu et al. 2019), water purification (Kumar et al. 2021), human nutrition (Aro et al. 2010; Dairo 2011), construction materials (Chimmaobi et al. 2020; Nwa-David et al. 2023; Ogunbode et al. 2023b; Olatokunbo et al. 2018; Raheem et al. 2020; Salau et al. 2012), biocomposites (Jullanun and Yoksan 2020; Martinaud et al. 2024; Ogunbode et al. 2023b; Versino et al. 2015; F. Zhu 2015), metal matrix composites (MMCs) (Edhirej et al. 2017a, b; Oladele et al. 2020; Olaniran et al. 2021), packaging materials (Thathsaranee et al. 2023; Tumwesigye et al. 2016), detoxifiers (Panghal et al. 2021; Tewe 1992), bioremediation (Adesiji and Ademola 2019; Adiaha 2021; Edeh et al. 2014; Kayiwa et al. 2021b), and supercapacitor electrodes for renewable energy (Ismanto et al. 2010; Taer et al. 2022; Versino et al. 2015).

### Bioenergy generation

Bioenergy, derived from organic agricultural waste, is a renewable energy source that can be converted into biofuels for transportation and biopower for heat and electricity generation. It currently accounts for 55% of global renewable energy and has the potential to achieve net-zero emissions, potentially displacing fossil fuels by 2030 and contributing to Sustainable Development Goal 7 for accessible and clean energy (Bains et al. 2023; UN 2015; 2023). However, achieving near-zero emissions remains challenging as bioenergy production releases the carbon initially sequestered by crops.

*Manihot esculenta* peels represent a significant bioenergy source, with literature documenting their potential in diverse applications, from household energy to aerospace (Adebisi et al. 2019; Andrade et al. 2021; Edhirej et al. 2021; Okudoh et al. 2014; Zhu et al. 2015). MEP can be harnessed for bioenergy through several processes, including anaerobic digestion to generate biogas, fermentation for bioethanol production, combustion or gasification for biopower, and pyrolysis for biochar. Bioenergy integration is advancing in industries such as aviation, where biofuels are used in a blend with jet fuels at a 30% mix (Sarisky-Reed 2016; Ahlgren 2002). Biopower has also contributed approximately 8% of energy to the electric grid (Kirchner and Thrän, 2019). The high carbon content of MEP makes it an excellent candidate for bioenergy applications, contributing to energy needs and supporting sustainable practices through waste valorization. These methods, discussed in subsequent sub-sections, offer renewable energy options ideal for rural areas where MEP is abundant and electricity access may be limited.

#### Biogas from *Manihot esculenta* peels

*Manihot esculenta* peels are a valuable feedstock for biogas production, providing renewable energy through methane and carbon dioxide output. Anaerobic digestion of MEP produces biogas with methane concentrations between 48 and 64%, suitable for heating, cooking, and electricity generation applications. However, MEP digestion faces challenges such as cyanide production and nitrogen deficiency, which can be addressed through digester innovations and co-digestion (Cuzin et al. 1992; Sirirote et al. 2010; Sirirote et al. 2010). The advantage of co-digestion lies in achieving balance in alkalinity, pH, and trace nutrients, contributing to higher biogas yields. Key findings and process conditions from related studies are summarized in Table 5, focusing on yield improvements, methane content, and optimization strategies.

**Table 5:** Biogas production from *Manihot esculenta* peels

Study	Method/conditions	Biogas yield and methane content	Remarks
Präger et al. (2019)	AD with MEP residues using stochiometric and substrate-specific methods	48.6–53% methane	Validated MEP as viable for bioenergy applications
Ismail et al. (2022)	Continuous digester at 35 °C for 25 days, at 0.076 gCOD.L^−1^ day^−1^ organic loading rate	~ 1000 mL biogas	Enhanced yield with controlled mesophilic digestion
Jaro et al. (2021)	Pig dung-optimized MEPs through AD	4.6–7.3 L/kg biogas, 0.73% methane	Optimized yield with specific substrate methods
Sirirote et al. (2010)	Co-digesting MEP with dried cow dung	64.3% methane and 13.20 L/day biogas	High methane content via co-digestion techniques
Aisien and Aisien (2020)	MEP-optimized cow dung slurry pretreated with NH_4_Cl	62.3% methane, 104,961 cm^3^ biogas	Enhanced methane and biogas yield with NH_4_Cl
Ofoefule and Uzodinma (2009)	Co-digestion with livestock dung	Increased yield for cattle (4.88 L), poultry (5.55 L), and swine (5.65 L)	Improved yield compared to the 2.29 L obtained using only MEPs

These studies emphasize that optimizing MEP digestion methods, especially through co-digestion, yields higher methane concentrations and stabilized pH levels. These advancements can be particularly beneficial in rural areas with abundant MEP resources and limited energy access.

#### Bioethanol from *Manihot esculenta* peels

**Fig. 6:**
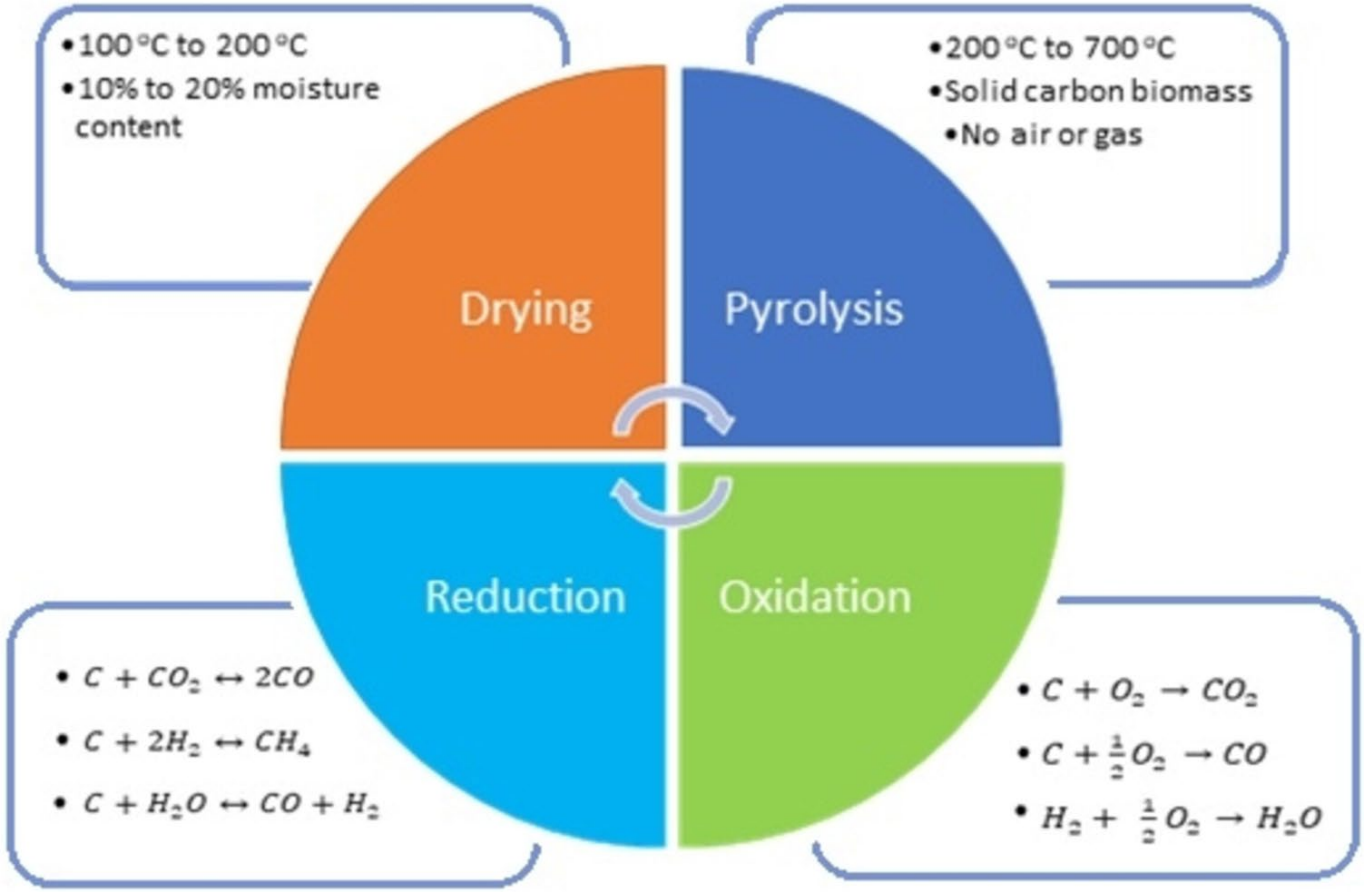
Flowchart of the gasification process

Bioethanol is a renewable biofuel produced via fermentation of sugars in plant residues, with *Manihot esculenta* peels serving as an efficient feedstock due to their high sugar content and favorable composition (Safari and Syafaat 2022). Figure 6 illustrates the thermochemical gasification process used to produce bioethanol from lignocellulosic biomass of lignin (C_9_H_10_O_3_(OCH_3_)_0.9–1.7_)_y_, hemicellulose (C_5_H_8_O_4_), and cellulose (C_6_H_10_O_5_) present in MEPs (Mukti et al. 2023). The production process involves soaking MEP to remove toxins, followed by drying, chopping, and pulverizing to enhance chemical interactions for hydrolysis, which is the breakdown of cellulose and hemicellulose into simple sugars (Adekunle et al. 2016; Chaoui and Eckhoff 2014). The sugars are then fermented into bioethanol, which can be distilled to obtain high-purity bioethanol.

The produced bioethanol can be extracted using the distillation extraction technique (DET) due to the scalability, high purity, energy efficiency, continuous operation, and co-product recovery of the DET. Carbon dioxide is subsequently removed in the form of gas vapor, with the unfermented sugar retrieved from the bottom of the distillation tank. The bioethanol is subsequently recovered as a vapor stream from the distillation column. For optimal bioethanol yield from *Manihot esculenta* peels, eleven factors must be considered for process optimization: Inoculum size (5.22%), alpha-amylase (24.74%), fermentation agitation speed (100 rpm), pre-hydrolysis temperature (50 °C), pre-hydrolysis time (24 h), pre-hydrolysis pH (pH 4), fermentation pH (pH 5), fermentation time (114 h), fermentation temperature (27 °C), substrate concentration (69.82 g/L), and the use of glucoamylase concentration (strongly recommended).

**Fig. 7:**
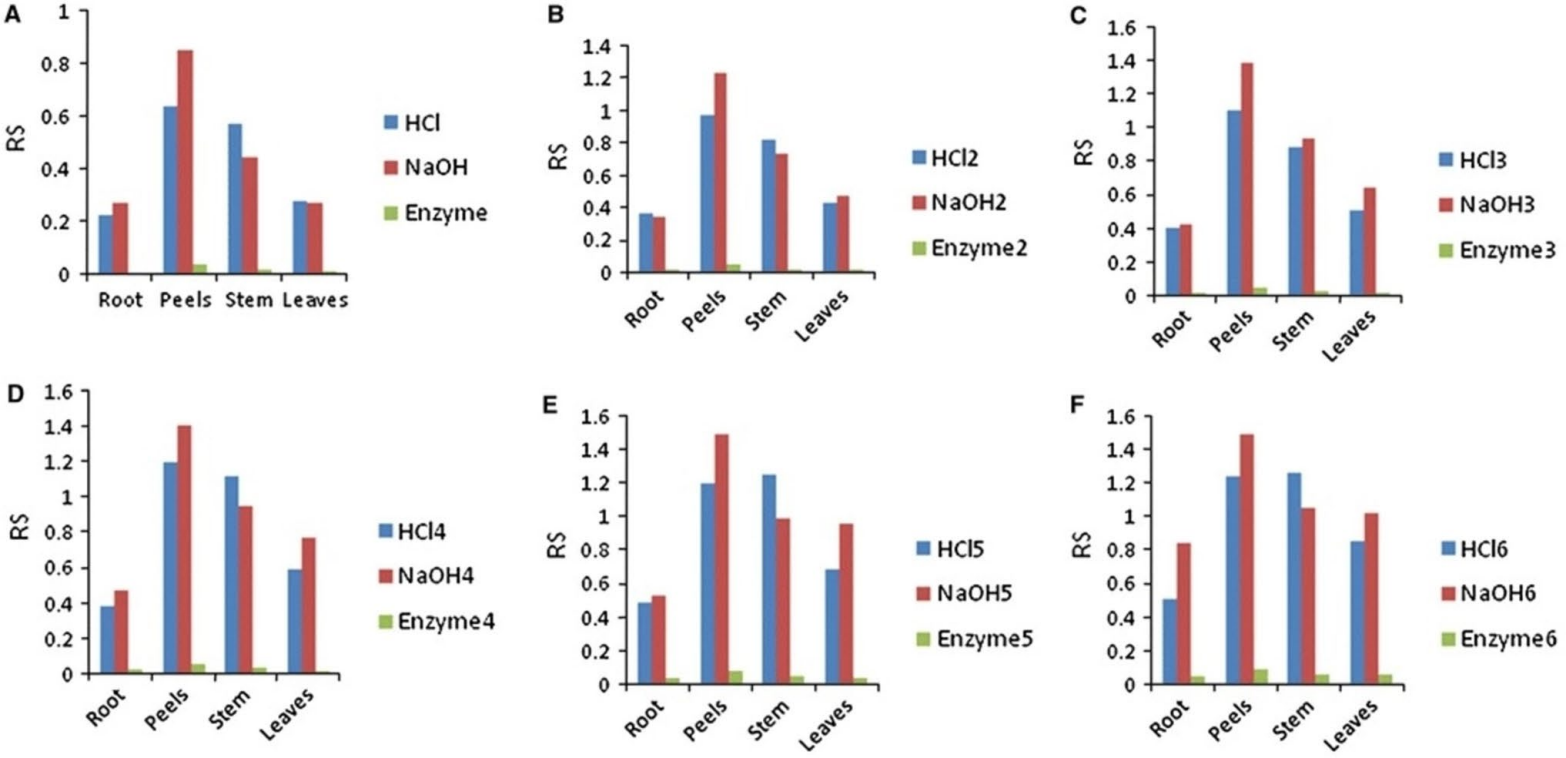
Result of hydrolysis of *Manihot esculenta* parts using acidic (HCl_x=1,2,.._), alkaline (NaOH_x=1,2,.._), and enzymes. Reproduced from Nuwamanya et al. (2012) under CC-BY

Key process optimization factors for high bioethanol yields include hydrolysis methods (acidic, alkaline, or enzymatic) and fermentation conditions such as temperature, pH, and time. These factors ensure the efficiency of bioethanol production, which can be enhanced through specific treatments and techniques like Simultaneous Fermentation and Saccharification (SFS) (Adekunle et al. 2016; Sivamani and Baskar 2015). The bioethanol produced can be used as a fuel or for other applications, including solvents and feedstocks for pharmaceuticals, with blends like E10 and E85 offering pollution-free combustion alternatives to gasoline (Anekwe et al. 2023; Khan et al. 2023; Segovia-Hernández et al. 2022; Sindhu et al. 2019). Research has demonstrated the high potential of MEP as a bioethanol feedstock compared to other energy crops residues like sugarcane, sweet sorghum, rice, maize, and wheat, as seen in Table 6, with examples showing bioethanol yields from MEPs as high as 69.82 g/L when using liquefaction and SFS methods. In addition, among the different parts of *Manihot esculenta*, MEPs yield the highest reducing sugars among different parts of *Manihot esculenta*, as shown in Fig. 7 (Nuwamanya et al. 2012). This makes them especially suitable for fermentation, leading to high bioethanol yields.

**Table 6:** Bioethanol production from *Manihot esculenta* peels

Study	Methodology	Bioethanol yield and characteristics	Remarks
Okudoh et al. (2014)	Energy crop residue comparison	6 t/ha yr of MEP, highest among crops	High bioethanol yield from MEP compared to other crops
Nuwamanya et al. (2012)	Hydrolysis (acidic, alkaline, enzymatic)	59.5–61.4% bioethanol, high reducing sugars	MEPs produce the highest yield of reducing sugars, which is desirable for fermentation
Ziska et al. (2009)	Field-based study (Alabama, Maryland)	55% bioethanol from MEP	MEPs show promising yields even in diverse environments
Sivamani and Baskar (2015)	Liquefaction and SFS	69.82 g/L bioethanol	Optimized fermentation and SFS for enhanced yield

These studies collectively highlight the significant potential of MEP as a feedstock for bioethanol production, demonstrating high yields and the need for optimized processing methods, particularly with alkaline hydrolysis and efficient fermentation techniques. However, bioethanol production faces other drawbacks beyond feedstock utilization. One such significant issue is the requirement for large cultivation areas, which can pose land use and agricultural sustainability challenges. In addition, the use of nitrogen fertilizers can contribute to eutrophication, an environmental concern associated with excessive nutrient runoff into water bodies (Andrade et al. 2021). Interestingly, the inherent composition of MEPs provides some advantages in this context, further enhancing their suitability as feedstocks for bioethanol production. Furthermore, MEPs may require less land for cultivation compared to other feedstocks, potentially mitigating concerns related to land use. Moreover, *Manihot esculenta* peels can thrive in various environmental conditions, thus reducing the reliance on nitrogen fertilizers and minimizing the risk of eutrophication.

#### Biochar from *Manihot esculenta* peels

Biochar is a sustainable material with high carbon content that is produced by heating organic waste in the absence of oxygen. It is resistant to decomposition due to its fine granularity and varied chemical surface properties, which can be hydrophilic, basic, acidic, or hydrophobic. This diversity in chemical properties contributes to its high reactivity potential. Biochar has diverse applications, including heat production, soil remediation, medical uses, metallurgy, and flue gas cleaning (Weber and Quicker 2018). Biochar is particularly notable for its greenhouse gas emissions. Its use can significantly reduce the emission of N_2_O (nitrous oxide), CO_2_, and CH_4_ (methane) by altering soil microbial activity, thereby decreasing anaerobic processes (Mosa et al. 2023; Shrestha et al. 2023; Zhou et al. 2023). These properties make biochar an effective tool in mitigating climate change.

Another crucial application of biochar is carbon sequestration. Biochar’s high carbon content enables it to capture and store carbon when added to soils, inhibiting the dispersion of CO_2_ into the atmosphere (Kumar et al. 2023). This long-term sequestration helps in reducing the overall concentration of greenhouse gases. Biochar’s highly porous structure enhances its suitability as a soil conditioner. It improves soil fertility, structure, and water retention capability (Odeyemi et al. 2023). These properties make biochar beneficial for agricultural productivity, especially in degraded soils. Biochar has also been used to remove phenol from industrial waste with a removal efficiency of about 82.3% (Emenike et al. 2022). These applications demonstrate biochar’s potential as a valuable tool in wastewater treatment, sustainable agriculture, and environmental remediation.

Several studies have explored the potential of using *Manihot esculenta* peels as feedstocks for biochar production (Anas et al. 2022; da Silva et al. 2022; Egbosiuba 2022; Grema et al. 2023; Li et al. 2018; Odeyemi et al. 2023). These investigations, summarized in Table 7, indicate that pyrolysis is the most commonly used method for producing biochar, with yields ranging from 24.60 to 68.59%. At lower pyrolysis temperatures, biochar yield was higher and reduced with temperature increase (Table 7). The higher biochar yields at lower temperatures are due to partial or incomplete pyrolysis of MEPs (Angın et al. 2013). Conversely, the rapid lignocellulosic decomposition of *Manihot esculenta* peels at higher temperatures increases volatile material production, which impacts the secondary decomposition of biochar residues, resulting in a lower yield (Hasan et al. 2019).

**Table 7:** Summary of studies investigating the use of *Manihot esculenta* peels as biochar produce

Method	Biochar yield (%)	Temperature (°C)	pH	Surface microstructure	Elemental composition (wt%)	Ash content (%)	TGA	FTIR	Source
Pyrolysis	68.59 to 56.92	300 to 600	2.35 to 4.96	Formation of pores and voids, homogenous distribution, rough surface. As temperature increases, pores, porosity, and surface area are enhanced, and micropores are formed	C(55.44–50.54), O(39.76–31.87), H(5.58–5.05), and N(3.56–1.11)	3.50 to 7.00	Thermal decomposition increased with pyrolysis temperature	C–H (670 cm^−1^ and 1400 cm^−1^), C = O (1650 cm^−1^), C≡C (2357 cm^−1^), O–H (3200 and 3490 cm^−1^)	(Egbosiuba 2022)
Pyrolysis	Not investigated (NI)	750	9.55	Enhanced surface area and large presence of micropores	C(62.38), H(1.52), and N(1.23)	30.56	NI	NI	(Li et al. 2018)
Pyrolysis	68.51	400	10	Rough surfaces, non-uniform pore distribution, and high surface porosity	C(78.65%), O(18.65%), P(0.26%), K(1.91%), Ca(0.30%), Mg(0.13%), Si(0.10%), and N(1.14%)	11.28	NI	O–H (745 and 3772 cm^−1^), C-H (872 cm^−1^), and C-O (1000 to 1400 cm^−1^)	(Grema et al. 2023)
Pyrolysis using a non-electrical reactor	55.13	338	NI	The surface is rough and porous, homogenous surface, non-uniformly distribtuted micropores	C(56.93), Ag(22.97), O(8.99%), N(5.81%), Ca(2.17%), Si(1.29), Al(1.12), and P(0.69)	NI	Improved thermal efficiency	OH, oxygenated group, aromatic C = C	(Odeyemi et al. 2023)
Pyrolysis	36.7 to 24.6	150 to 450	5.26 to 9.05	NIdistributed	Not investigated	NI	Reduced thermal efficiency as more biomass was converted to gases and volatiles	OH (3425 cm^−1^), C = C (1625 cm^−1^), and oxygenated group (1389 cm^−1^) band	(da Silva et al. 2022)
Pyrolysis	NI	300	NI	The microstructure shows a porous surface with irregular pores	C(70.22) and O(29.78)	NI	NI	-OH (3256 cm^−1^), C = C (1554 cm^−1^), and C-O (1034 cm^−1^)	(Anas et al. 2022)

**Fig. 8:**
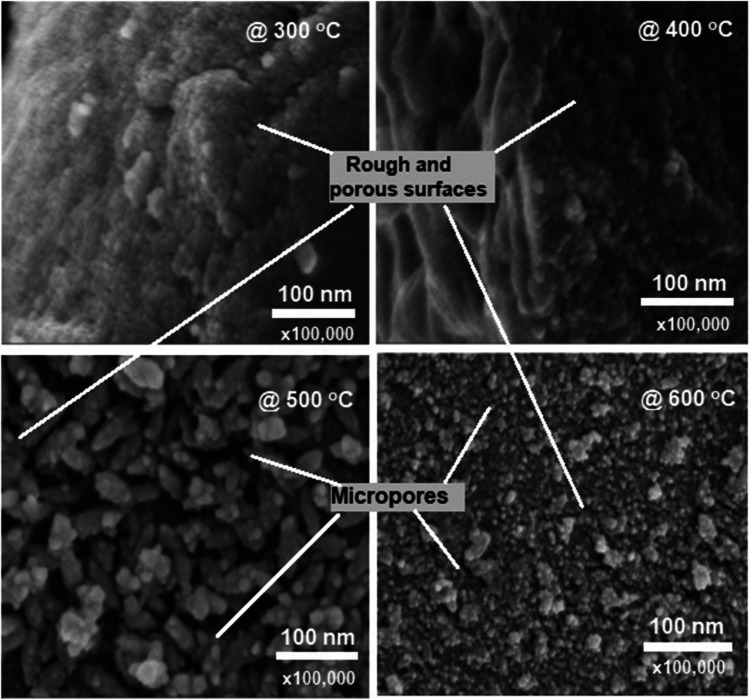
Surface micrograph of MEP-based biochar at different temperatures. Modified from (Egbosiuba 2022) under CC-BY

Microstructure investigations of MEP-based biochar consistently show that as temperature rises, uniformly distributed micropores with increased porosity are formed (Fig. 8). The porous properties of MEPs have been experimentally validated by (Ben and Olubambi 2024b), showing that mesopores and micropores are produced through the carbonization process of MEPs. The rough and porous surfaces make MEP-based biochar effective as absorbents and for soil remediation. Additionally, MEP-based biochar’s pH rises with increasing pyrolysis temperature (da Silva et al. 2022) and is useful for reducing soil acidity and enhancing nutrient availability (Al-Wabel et al. 2018; Lehmann et al. 2011; Shi et al. 2019). All the studies assessed in Table 4 reveal that carbon is the most dominant element in MEP-based biochar, with a maximum value of 78.65% observed by (Grema et al. 2023). Other elemental compositions observed in MEP-based biochar include O, H, N, Ca, silver (Ag), and trace amounts of Si, Al, P, K, and Mg. Fourier Transform Infrared (FTIR) spectroscopic investigations confirmed the existence of hydroxyl, aromatic, and oxygenated functional groups at varying band peaks influenced by the pyrolysis temperatures. Thermogravimetric analysis (TGA) indicates that thermal efficiency is a function of both the pyrolysis temperature (Egbosiuba 2022) and the amount of biomass used for biochar production (da Silva et al. 2022).

Despite the significant benefits associated with high-yield biochar production using *Manihot esculenta* peels, several drawbacks have been identified from reviewed experimental studies. These include high ash content (Anas et al. 2022; Andiani et al. 2022), high moisture content (Rudiyanto et al. 2023), presence of toxic compounds (Aso et al. 2018), limited surface area (Anas et al. 2022), nutrient imbalance (Andiani et al. 2022; Odeyemi et al. 2023), and high production costs (Odeyemi et al. 2023). These drawbacks have been shown to limit the biochar yield from MEPs compared to other materials. For instance, the limited surface area of MEP-based biochar impacts its adsorption properties (Anas et al. 2022; Odeyemi et al. 2023). Among the different ME parts, biochar produced from the ME peels has a limited surface area due to fewer functional groups (Mopoung et al. 2012). However, the surface area can be enhanced through an improved activation process, which increases micropores (Anas et al. 2022; Mopoung et al. 2012). Optimizing pyrolysis temperature can effectively address the high ash content, as seen in Table 7, where lower pyrolysis temperatures result in higher biochar yields.

High moisture content can be mitigated by ensuring *Manihot esculenta* peel biomass is well-dried before pyrolysis. Cyanogenic glycosides (toxic compounds) in MEPs necessitate further studies to understand their impact on biochar yields. However, proper pre-treatment techniques like soaking and appropriate drying methods can effectively reduce cyanide content (Ben and Olubambi 2024a). The high potassium content in MEPs can lead to ash-related issues and negatively impact the quality of the produced biochar (Andiani et al. 2022). For instance, a 10.8 wt% potassium value was observed for MEPs by (Ben and Olubambi 2024b). High potassium values can result in nutrient imbalances in soils where MEP-based biochar is applied. Blending MEPs with other nutrient-rich biochars, such as poultry litter or animal droppings, is recommended to address drawbacks associated with nutrient imbalance. Regarding production costs, a relatively cheap and environmentally friendly non-electrical reactor has been proposed and demonstrated by (Odeyemi et al. 2023), with results consistent with existing studies.

### Biocomposites

Synthetic polymers face challenges like insufficient organic matter, environmental pollution, non-biodegradability, consumer toxicity risks, and cross-contamination issues (Nagalakshmaiah et al. 2019; Siracusa et al. 2008), biocomposites offer a sustainable solution. Biocomposites are composite materials that use biodegradable polymers as the matrix, reinforced by natural fibers primarily derived from residues of agricultural wastes. The utilization of agro-waste residues in biocomposites is driven by their natural abundance and the need to address environmental concerns associated with pollution from these wastes. Reinforcing biopolymers with agro-waste residues enhances mechanical strength and thermal stability and provides a sustainable strategy for managing agricultural waste (Benito-González et al. 2019; Lomelí Ramírez et al. 2011).

**Fig. 9:**
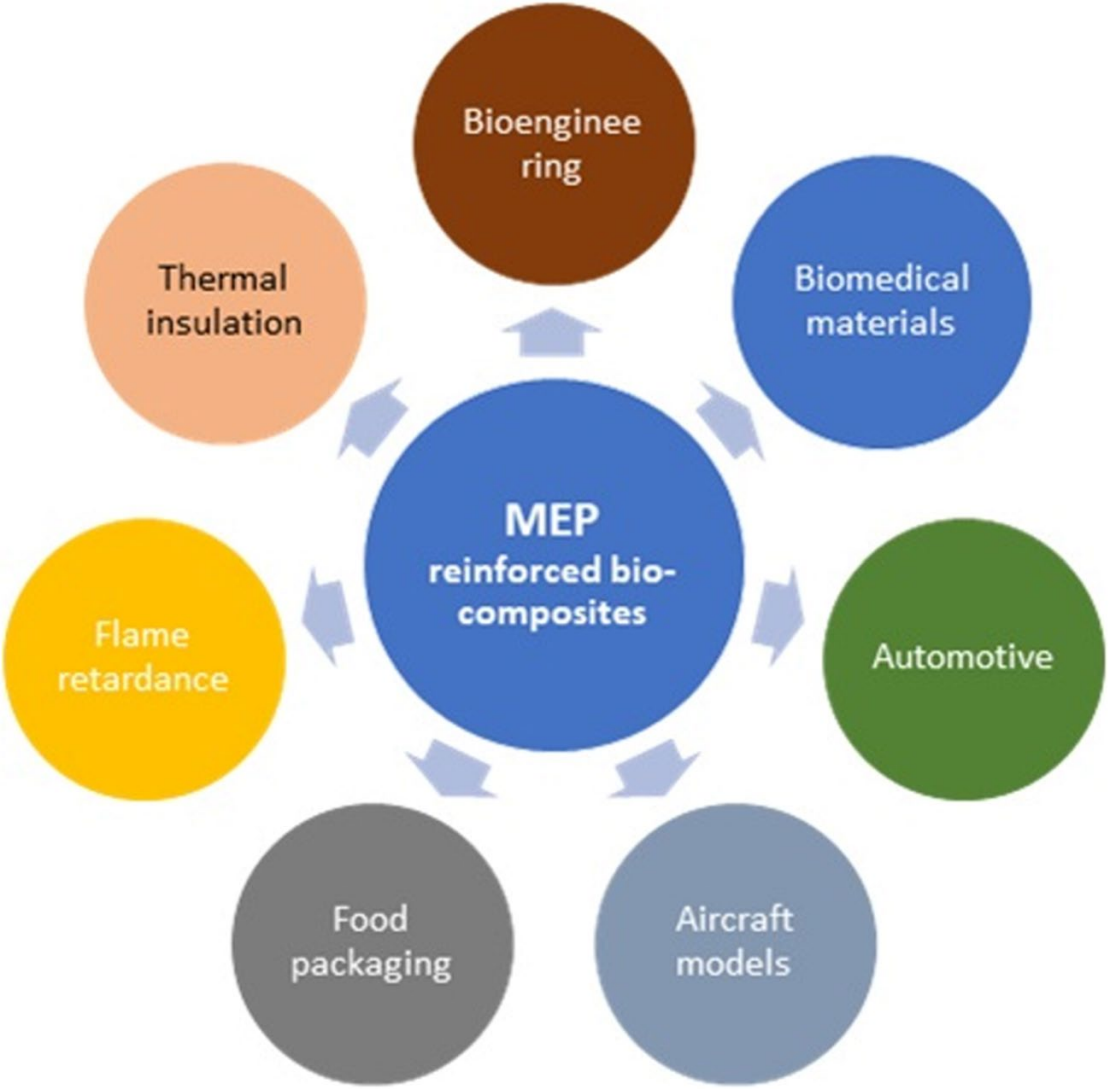
Potential application areas of MEPs fiber-reinforced biocomposites

Biocomposites have found diverse applications (Fig. 9), including bioengineering (Huang 2023), biomedical materials for hard tissues, soft tissues, and implants (Ramakrishna and Huang 2016), and as replacements for non-biodegradable petroleum-based materials (Nagalakshmaiah et al. 2019). They are also used in vehicle interior paneling, brake linings, canoes, aircraft models (Riedel 2012), and food packaging (Siracusa et al. 2008). Agro-waste residues like *Manihot esculenta* peels, rice husk, and livestock droppings have been researched to develop innovative bio-based materials to mitigate greenhouse gases by lowering carbon dioxide emissions to the atmosphere. Biocomposites reinforced with agro-wastes have been shown to exhibit enhanced mechanical properties, improved electronic performance, superior wear resistance, flame retardance, and thermal insulation (Koronis et al. 2013; Nagalakshmaiah et al. 2019).

Studies on the use of *Manihot esculenta* bagasse (MEB) as reinforcing filler agents have been extensively documented, driven by the high starch content in this category of food crop (Edhirej et al. 2021; Edhirej et al. 2017a, b; Edhirej et al. 2017a, b). However, there has been limited research on using *Manihot esculenta* peels for the same purpose despite evidence suggesting that the crude fiber content in MEPs surpasses that in other parts of the plant (Abotbina et al. 2022; Ben and Olubambi 2024b). For instance, MEPs contain 10.6% crude fiber compared to only 2.0% in MEB (Marx and Nquma 2013). Similarly, dried MEPs have a higher cellulose content, whereas MEB contains about 38% cellulose (Versino et al. 2015). Cellulose from MEPs is crucial in biocomposite fabrication due to its biodegradability, high tensile strength, stiffness, lightweight, compatibility, thermal stability, abundance, and cost-effectiveness. Cellulose nanofibers, extracted from starch-based food crops, are used to reinforce biocomposites for use in the production of high-strength materials, eco-friendly packaging, biomedical devices, and filtration systems. Table 8 provides examples of studies using *Manihot esculenta* peels as a reinforcement material in biocomposites, demonstrating the effectiveness of various processing methods.

**Table 8:** Selected studies on *Manihot esculenta* peel as reinforcement in biocomposites

Study	Processing method	Key findings	Potential applications
Leite et al. (2017)	Acid hydrolysis	Produced high-crystallinity nanofibers with diameters of 2.3–5.4 nm	Biomedical materials, high-strength goods
Travalini et al. (2019)	Alkali treatment, bleaching	Created cellulose nanofibers with improved thermal stability with mean diameters of 6.7 nm and 8.2 nm	Food packaging, eco-friendly bioplastics
Nwiyoronu et al. (2022)	Soil burial test	Enhanced biodegradability and adjusted water absorption properties	Biodegradable packaging
Versino et al. (2015)	FTIR, microstructural analysis	Improved thermodynamic properties of TPS biocomposites with enhanced opacity and UV-blocking	Thermoplastic starch products, UV shields
Thathsaranee et al. (2023)	Crystallinity phase analysis	Found α-amylose dihydrate, enhancing stability and thermal properties	Sustainable packaging materials

**Fig. 10:**
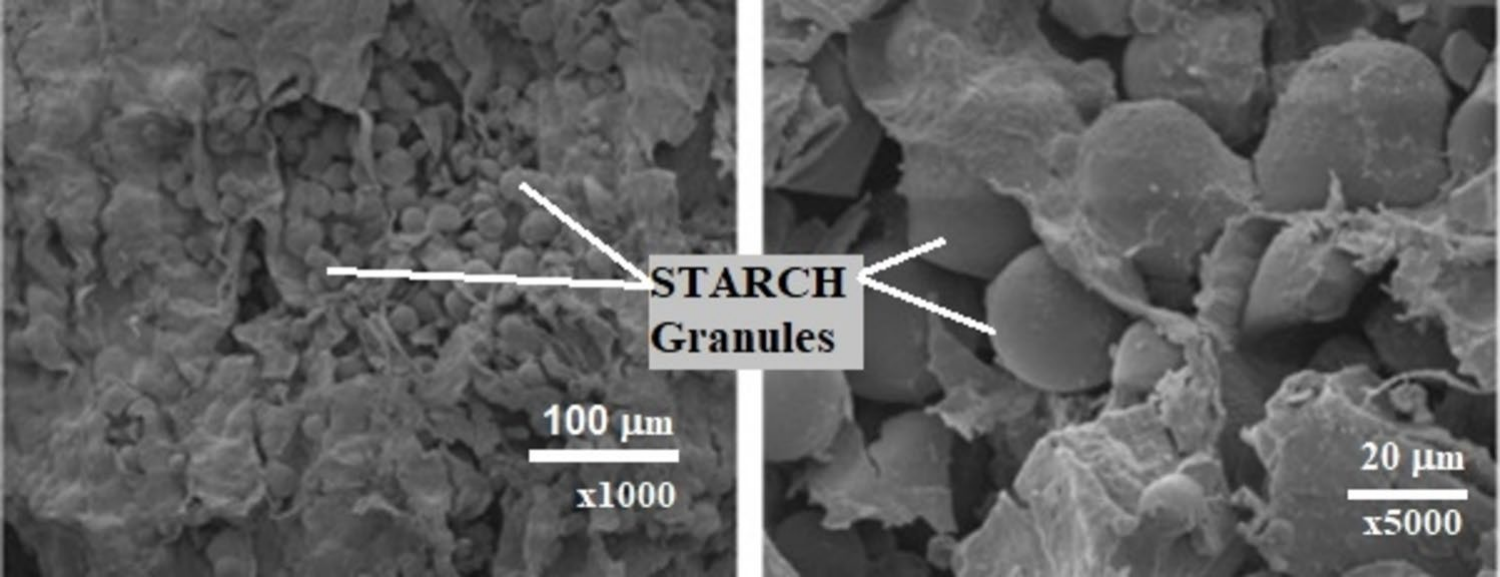
Surface morphology of MEP-reinforced TPS biocomposite. Reproduced from (Versino et al. 2015) under CC-BY

These studies suggest that the presence of lignin and protein in MEPs can enhance biocomposites’ mechanical stability and strength. Chemical and physical modifications can also boost the mechanical strength of biocomposites (Czaikoski et al. 2020), while pre-conditioning methods optimize starch gelatinization to achieve effective barrier characteristics (Benito-González et al. 2019). Incorporating MEPs reduces ductility but enhances properties such as tensile and flexural strength, influenced by factors like filler-matrix adhesion (Fig. 10), processing, and filler treatment (Ben and Olubambi 2024b; Gomes et al. 2007). Additionally, the low density of MEPs makes them suitable for lightweight applications, including aerospace technology. MEPs also contribute to biocomposites’ biodegradability and water absorption, with soil burial tests confirming a steady decline in biocomposite weight over time (Nwiyoronu et al. 2022). FTIR analysis has shown that lignin and protein in MEPs enhance mechanical stability, while MEPs’ phenolic compounds improve UV absorption and opacity (Chaurasia and Lal 2016). Furthermore, the reduced transparency and increased opacity from larger MEP particles indicate their potential in applications such as biodegradable food packaging, where cost-effectiveness, non-toxicity, and durability are essential.

Some drawbacks in using *Manihot esculenta* peels for biocomposite production include high ash and moisture content, toxicity, and compatibility issues. MEPs’ high ash and moisture content can negatively impact the produced biocomposite’s mechanical performance and thermal stability as a result of poor interfacial bonding between fibers and the matrix. Additionally, cyanide compounds present in MEPs can make the biocomposite toxic, posing significant health risks, especially in applications like food packaging and car seats. Effective pre-treatment techniques can address high ash and cyanide content challenges, while thorough drying of the MEPs before use will mitigate the moisture content problem. Chemical treatments have been explored to reduce moisture susceptibility (Azwa et al. 2013). Incompatibility issues between fiber and matrix can reduce the mechanical strength of the produced biocomposite arising from the hydrophilic nature of natural fibers, with the likelihood of forming aggregates during processing, which limits their processing temperatures (Versino et al. 2015). Therefore, it is imperative to grasp natural fibers’ inherent compositions and characteristics to be used as polymer fillers. FTIR, XRD, SEM, TGA, and crystallinity tests are crucial for assessing the suitability of natural fibers for biocomposite production.

### Building and construction materials

Cement is an essential material peculiar to the building and construction industries. However, it has been identified as a significant carbon dioxide emitter, accounting for ~ 8% of global carbon dioxide emissions, compared to 2.5% from aviation fuel (Rodgers 2018). China is the leading cement producer worldwide at ~ 2.1 billion metric tons, with India and Vietnam at ~ 410 and ~ 110 million metric tons, respectively (Jaganmohan 2024; Tkachenko et al. 2023). The manufacturing process, particularly during the clinker production at the calcination stage, contributes significantly to greenhouse gas emissions, at approximately 7% (Miller et al. 2021). This environmental impact has spurred a search for environmentally friendly and sustainable construction materials to mitigate the detrimental effects of conventional construction materials. Agro-wastes has emerged as a promising alternative binder in cement and concrete formulations, offering a dual benefit of effective waste management and the development of “green cement” (AlBiajawi et al. 2022; Amare et al. 2023; He et al. 2020; Manikanta et al. 2020). This green cement provides a sustainable, eco-friendly alternative to Ordinary Portland Cement (OPC), with a carbon capture potential that highlights the valorization of MEPA in sustainable cement production. Studies demonstrate that MEPA exhibits high pozzolanic properties, especially when calcined under specific conditions (Salau and Olonade 2011; Aliu et al. 2023). MEPA’s oxide composition, which is rich in K₂O, SiO₂, MgO, and Al₂O₃ (Table 4), meets the ASTM C618 standards, with the required trioxide totals of Fe₂O₃, Al₂O₃, and SiO₂ expected to have a minimum of 70% for Class N, and 50% for Classes F and C (Akinkunmi and Olanrewaju 2018; Olatokunbo et al. 2018; ASTM 2023).

*Manihot esculenta* peel ashes as a supplementary concrete additive in the building and construction industries have been extensively studied (Table 9). This exploration is driven mainly by the rising costs of pozzolans, whose production costs have increased significantly over the years (Olatokunbo et al. 2018). MEPA’s pozzolanic potential has been found to enhance compressive strength and durability, making it an effective supplementary cementitious material. For example, in comparative studies (Table 9), MEPA showed a compressive strength of 14.87 N/mm^2^ after 7 days, slightly surpassing RHA (rice husk ash) and PSA (periwinkle shell ash) alternatives, indicating its robust mechanical performance (Akinkunmi and Olanrewaju 2018). When applied in biocomposites, MEPA’s oxide-rich composition contributes to reduced environmental impact by partially substituting OPC, aligning with ASTM standards for environmentally friendly construction materials. The chemical characteristics of MEPA make it viable for lightweight applications requiring high strength and stiffness, suitable for a range of engineering uses.

**Table 9:** The use of *Manihot esculenta* peels as potential cement replacements

Weight ratios	Slump	Compaction factor	Density	Compressive strength	Tensile splitting strength	Water absorption	Porosity properties	Ref	Remark
5, 10, 15, 20, and 25%	Decrease with increasing wt%	Increases with increasing MEPA wt%	Ranged between 2410 and 2493 kg/m^3^	Decreases with increasing MEPA content but increases with age curing	Decreases with increasing MEPA content but increases with age curing	Increases with rising MEPA values and curing ages	Reduces with curing ages and increases with rising MEPA wt%	(Olatokunbo et al. 2018)	The recommended replacement weight ratio is 10% MEPA
5, 10, 15, 20, and 25%	Decreases with increasing wt%	NI	NI	Increases with curing age but decreases with increasing wt%	Increases with curing age, decreases with rising MEPA wt%	NI	NI	(Eziefula et al. 2019)	15% MEPA replacement is recommended
5, 10, 20, 30, and 40%	Decreases with increasing wt%	Increases with increasing MEPA wt%	NI	Rises with c0075ring and decreases with increasing weight ratios	Rises with curing and decreases with increasing weight ratios	Increases with rising wt%	Reduced surface area with rising wt%	(Chimmaobi et al. 2020)	10% recommended MEPA replacement
5, 10, 15, 20, and 25%	NI	NI	NI	Increases with curing age but decreases with increasing MEPA particles	NI	Decrease with an increase in the percentage MEPA replacement	Improved pore structure with rise in wt%	(Abdulwahab and Uche 2021)	5% MEPA replacement is recommended
5, 10, 15, 20, and 25%	Decreases with an increase in the amount of MEPA	Variation decreases with increasing MEPA wt%	Ranged between 2414 and 2473 kg/m^3^	Increases with age and reduces with increase in MEPA content	Decreases with rising MEPA content	NI	NI	(Salau et al. 2012)	15% MEPA replacement is recommended
5, 10, 15, and 20%	Decreases with increase in MEPA wt%	Increases with increase in MEPA wt%	Not investigated	Increased with length of curing age, but decreased as the percentage of MEPA increases	NI	NI	NI	(Raheem et al. 2020)	15% MEPA replacement is recommended
10, 20, 30, and 40%	Decreases with increase in MEPA wt%	NI	Ranged between 2204 to 2537 kg/m^3^	Decrease with an increase in MEPA wt% and increases with curing age	NI	High water absorption	NI	(Ogunbode and Akanmu 2012)	30% MEPA replacement is recommended

The results of Tables 4 and 9 suggest that integrating agro-waste particles like MEPA in cement production can reduce costs and minimize greenhouse gas emissions, but only as partial replacements in pozzolanic production. However, it should be noted that this green cement, despite the promising results, should be limited to lightweight concrete production, as there is currently no evidence to support or recommend their use as complete cement replacement in the building and construction sectors. Furthermore, extensive studies are still required to be done with strict adherence to the ASTM C1709-22 standard guide (ASTM 2022), as all of the studies assessed in Table 9, except for (Olatokunbo et al. 2018), did not fully comply with the ASTM C1709 test guides for alternative cementitious materials as many important test validations were not carried out, making it difficult to ascertain the potential of MEPA particulate replacements in concretes.

### Supercapacitor electrodes for renewable energy

Supercapacitor electrodes are essential components of supercapacitor batteries used for energy harvesting and storage in renewable energy applications. These devices can store energy through electrochemical processes and release it through electrostatic discharge much faster than regular batteries. Supercapacitors are gaining rapid application in electric and hybrid vehicles, portable and wearable devices, power grids, renewable energy systems, robotics, military devices, and telecommunication base stations. Their exceptional performance is attributed to a broad temperature tolerance, elevated power density, extended life cycle, and swift discharging and charging times (González et al. 2016; Kandalkar et al. 2010; Meng et al. 2017). The nature of electrode material is fundamental in determining a supercapacitor’s performance characteristics. Commonly used materials as supercapacitor electrodes include manganese, ruthenium, and nickel metal oxides (Song et al. 2012; Yang et al. 2014), polypyrrole and polyaniline conducting polymers (Deng et al. 2017; Meng et al. 2017), and carbon-based materials (activated carbon, graphene, and CNTs) (Andiani et al. 2022; Ismanto et al. 2010; Kayiwa et al. 2021b; Sudaryanto et al. 2006; Yang et al. 2015; Zou et al. 2018).

**Fig. 11:**
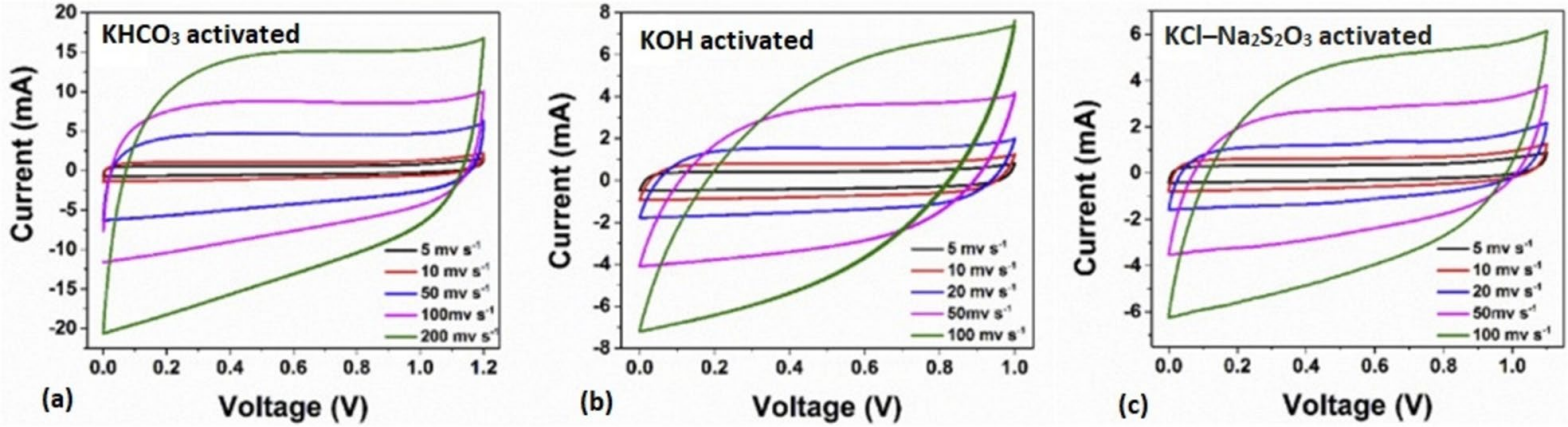
Cyclic voltammogram of MEPA particulate-based electrodes activated KHCO_3_, KOH, and Na₂SO₄ electrolytes. Modified from (Amakoromo et al. 2021) under CC-BY

Activated carbon is largely adopted for electrode production as a result of its high surface area, chemical stability, good conductivity, environmental abundance and sustainability, and compatibility with diverse electrolytes (Ismanto et al. 2010; Sevilla and Mokaya 2014). Although CNTs and graphene are also widely utilized for their excellent conductivity and mechanical strength (Afzal et al. 2017; Yang et al. 2015). However, they are less cost-effective than activated carbon, mostly obtained from agro-waste sources (Wang et al. 2020). Examples of agro-wastes studied for producing activated carbon for supercapacitors include corn cob ash (Yang and Zhang 2018), rice husk ash (Zhong et al. 2018), sugarcane bagasse ash (Zou et al. 2018), coconut shell ash (Xia et al. 2018), orange peel ash (Ranaweera et al. 2017), and *Manihot esculenta* peel ash (Amakoromo et al. 2021). Studies utilizing MEPA as electrode materials (Table 10) have shown excellent porosity and rough surface areas, with activated carbon significantly enhancing the performance of these MEPA-based carbon electrodes for surface modifications. Furthermore, carbon samples from MEPA have been shown to enhance ionic reactions during the electrochemical process, with the cyclic voltammogram result, as seen in Fig. 11 at 5 to 10 mV/s scan rates.

**Table 10:** Performance analysis of MEPA-based supercapcitor electrodes

Study	Electrode material	Activating agent	Specfic capacitance	Remark
Ismanto et al. (2010)	MEPA	HNO₃, H₂O₂, and H₂SO₄	264.08 F/g	Surface area was not significantly modified but carbon surface chemsitry was enhanced
Amakoromo et al. (2021)	MEPA	KHCO₃, KOH, and a blend of KCl-Na₂S₂O₃	300 F/g at 0.5 A/g	98% capacitance retention, and 828 m^2^/g specific surface area with excellent porosity and rough surface areas
Taer et al. (2022)	MEPA	KOH, H₂SO₄, and Na₂SO₄ aqueous electrolytes	112 F/g (Na₂SO₄), 150 F/g (KOH), and 183 F/g (H₂SO₄) at 1 mV/s scan rate	The fabricated electrodes were found suitable for applications requiring frequent cycling, with an equivalent series resistance (ESR) of 0.21 to 0.42 Ω and Coulombic efficiency of 89%
Ospino et al. (2020)	MEPA	KOH and H₃PO₄	64.18 F/g	Cyclic voltammetry potential ranging from −0.4 V to 0.6 V, with 398.46 m^2^/g specific surface area was obtained
Harahap et al. (2022)	MEPA	Chemical impregnation techniques with 94.61% carbon and 2.67% self-doped oxygen	257 F/g at 1 A/g	High-performance electrode material with Coulombic efficiency of 91.10% observed at 10 A/g

The use of *Manihot esculenta* peels as potential raw materials for producing electrodes in supercapacitors has its drawbacks. For instance, the quality of MEPA particulates can affect processing conditions, leading to inconsistent properties in the fabricated activated carbon electrodes, as observed in various studies (Amakoromo et al. 2021; Ismanto et al. 2010; Taer et al. 2022; Ospino et al. 2020; Harahap et al. 2022). Furthermore, agro-waste biomass often exhibits irregular morphologies, which can affect pore properties and limit performance rate and power density (Wang et al. 2020). Therefore, industries adopting MEPA particulates must develop adequate chemical treatment techniques to minimize differences in chemical composition, surface area, and porosity. The incorporation of heteroatoms has been proposed to shorten the ion/charge transportation distance, thereby enhancing the properties of supercapacitor electrodes made from agro-wastes (Wang et al. 2020). In addition, MEPA-based activated carbons specific capacitance is relatively lesser than materials like graphene or CNTs, impacting their electrochemical performance and structural integrity.

There is also a need to research non-toxic activating chemicals to mitigate the side effects and hazardous nature of commonly used activating agents such as KOH, H₃PO₄, and H₂SO₄. The activation process is equally energy-intensive, which can increase the overall production cost. Therefore, further studies should explore strategies to reduce the environmental impact associated with this process. Standardized methods for estimating the performance of agro-waste residues like MEPA particulates are required due to the wide variation in supercapacitor electrode performance reported. A supercapacitor cell typically has two electrodes, which can be identical or different, with a separator immersed in an electrolyte to prevent electrical contact between the electrodes (González et al. 2016). For enhanced measurement, one of these electrodes can be made from a reference carbon material with well-established surface area and capacitance values, while the second electrode can be made from MEPA particulates. This approach effectively assesses the potential of MEPA particulates in fabricating supercapacitor electrodes.

## Conclusion and future outlook

This study reviewed the waste management challenges associated with *Manihot esculenta* peels and presented innovative solutions to repurpose this agro-waste into valuable materials for sustainable engineering applications. The review explored heat treatment techniques like carbonization, calcination, and ashing, revealing promising applications for *Manihot esculenta* peels. Key findings include the following:

1. The oxide composition of MEPs makes them suitable for partial replacement of Portland cement due to their high pozzolanic properties, meeting ASTM standards for Classes N, F, and C.
2. MEPs’ smooth, non-porous surface suggests effectiveness as fillers in composites, enhancing mechanical properties for biocomposites, wastewater treatment, and soil conditioning.
3. MEPs’ high carbon content offers strong bioenergy potential, with yields exceeding other agro-waste residues like rice husk, corn cob, periwinkle, sugarcane, and coconut shell ashes, making them suitable for sustainable biogas, bioethanol, and biochar production.
4. MEP-based biodegradable biocomposites have excellent ductility, strength, and thermal stability, supporting aerospace, automotive, and lightweight construction applications.
5. Supercapacitor electrodes made from MEPs exhibit high porosity and specific capacitance, showing improved performance over electrodes from other agro-waste residues.

Despite extensive research on valorizing *Manihot esculenta* peels as from an environmental pollutant to sustainable engineering materials, future studies could address key limitations identified in the literature by considering the following:

1. Optimize pyrolysis temperatures for enhanced biochar production to reduce high ash content.
2. Additional research is needed to evaluate MEPA's potential as a reinforcing filler in hybrid and monolithic biocomposites for improved mechanical and electrochemical properties.
3. New processing techniques should be developed to address compatibility issues between MEPA-based fillers and matrix materials, preventing aggregates that impact mechanical properties.
4. Comprehensive studies should follow ASTM C1709 guidelines to validate MEPA particles as alternative cementitious materials in concrete applications.
5. For supercapacitor electrodes, standardized chemical treatment methods are essential to achieve consistent performance by minimizing chemical composition, surface area, and porosity variations in MEPA particulates.
6. Research is required on safe, non-toxic activating chemicals to reduce environmental risks in MEPA applications.
7. Standardized performance metrics for agro-waste residues like MEPA in supercapacitors would ensure reliable comparison across studies.
8. Solutions are needed for cyanide inhibition in MEP-based biogas production and for overcoming limitations in bioethanol yield due to high cellulose.
9. Innovations in processing can improve MEP-based biochar and biocomposites, making them more competitive with other agro-waste residues.
10. Increasing MEP replacement percentages in cement applications can reduce environmental impacts and enhance cost-effectiveness, necessitating rigorous studies to optimize pozzolanic properties.
11. Developing high-quality MEP-derived electrodes for supercapacitors is vital for renewable energy; further research should refine activation techniques to enhance electrode quality for energy storage systems.

Finally, the sustainable utilization of *Manihot esculenta* peels holds great potential for a cleaner and sustainable engineering future. Continued investigations and innovation are essential to unlock this potential and transform *Manihot esculenta* peels into a key resource for various engineering applications. By addressing the current challenges and optimizing processing techniques, *Manihot esculenta* peels can transition from being an environmental burden to a valuable resource. Increased and continued advancements in processing technologies and material science provide a veritable pathway for the broader application and optimization of *Manihot esculenta* peels in sustainable engineering solutions.

## Data Availability

No dataset was analyzed or generated as this study proceeds within a theoretical and mathematical approach.
